# Significance of serology testing to assist timely diagnosis of SARS-CoV-2 infections: implication from a family cluster

**DOI:** 10.1080/22221751.2020.1752610

**Published:** 2020-05-13

**Authors:** Yan Xu, Meng Xiao, Xinchao Liu, Shengyong Xu, Tiekuan Du, Jun Xu, Qiwen Yang, Yingchun Xu, Yang Han, Taisheng Li, Huadong Zhu, Mengzhao Wang

**Affiliations:** Peking Union Medical College Hospital, Chinese Academy of Medical Sciences and Peking Union Medical College, Beijing, People’s Republic of China

## Abstract

Confirmative diagnosis of SARS-CoV-2 infections has been challenged due to unsatisfactory positive rate of molecular assays. Here we identified a family cluster of SARS-CoV-2 infections, with five of six family members were SARS-CoV-2-specific immunoglobin serology testing positive, while molecular assays only detected two of this five patients even repeated twice. We comprehensively analyzed this familial cluster of cases based on the clinical characteristics, chest CT images, SARS-CoV-2 molecular detection results, and serology testing results. At last, two patients were diagnosed with COVID-19, two were suspected of COVID-19, and two were considered close contacts. Our results emphasized the significance of serology testing to assist timely diagnosis of SARS-CoV-2 infections, especially for COVID-19 close contacts screening.

Coronavirus disease 2019 (COVID-19) now have a trend of global spread, and had been declared as an international public health concern [1]. It was caused by a novel enveloped RNA betacoronavirus severe acute respiratory syndrome coronavirus 2 (SARS-CoV-2) [2]. Although fever and cough are the primary clinical presentations of COVID-19, fever is present in only 43.8% of patients on admission, which complicates initial clinical diagnosis [3]. In addition, 1.2% asymptomatic COVID-19 cases have been reported in China [4]. In current WHO recommendations [1] and China official guidelines, confirmative diagnosis of COVID-19 relies on SARS-CoV-2 molecular assays. However, the current strategy of SARS-CoV-2 molecular assays used for COVID-19 diagnosis is not perfect[5]. From our experience in a previous COVID-19 family cluster, significance of serology testing for the disease should be more emphasized.

On February 5, 2020, a 61-year-old female patient (Case 1) and her 64-year-old husband (Case 2) presented to the Fever Clinic of the Peking Union Medical College Hospital (PUMCH) for fever and respiratory symptoms. Case 1 and Case 2 previously lived in Wuhan, bringing their grandson (Case 5) with them, and three of them travelled to Beijing on January 22, to have family reunion for the Chinese New Year with their daughter’ family. Base on the epidemiological history and symptoms, real-time reverse-transcriptase–polymerase-chain-reaction (RT-PCR) assay of nasopharyngeal swab specimens for SARS-CoV-2 detection and chest CT scanning were performed for Case 1 and Case 2. Chest CT images of Case 1 (Figure 1a) showed bilateral ground-glass opacity and chest CT images of Case 2 (Figure 1b) showed bilateral patchy shadowing, both of which indicated viral pneumonia. However, SARS-CoV-2 RT-PCR testing result for Case 1 was positive, but negative for Case 2.

**Figure 1.**
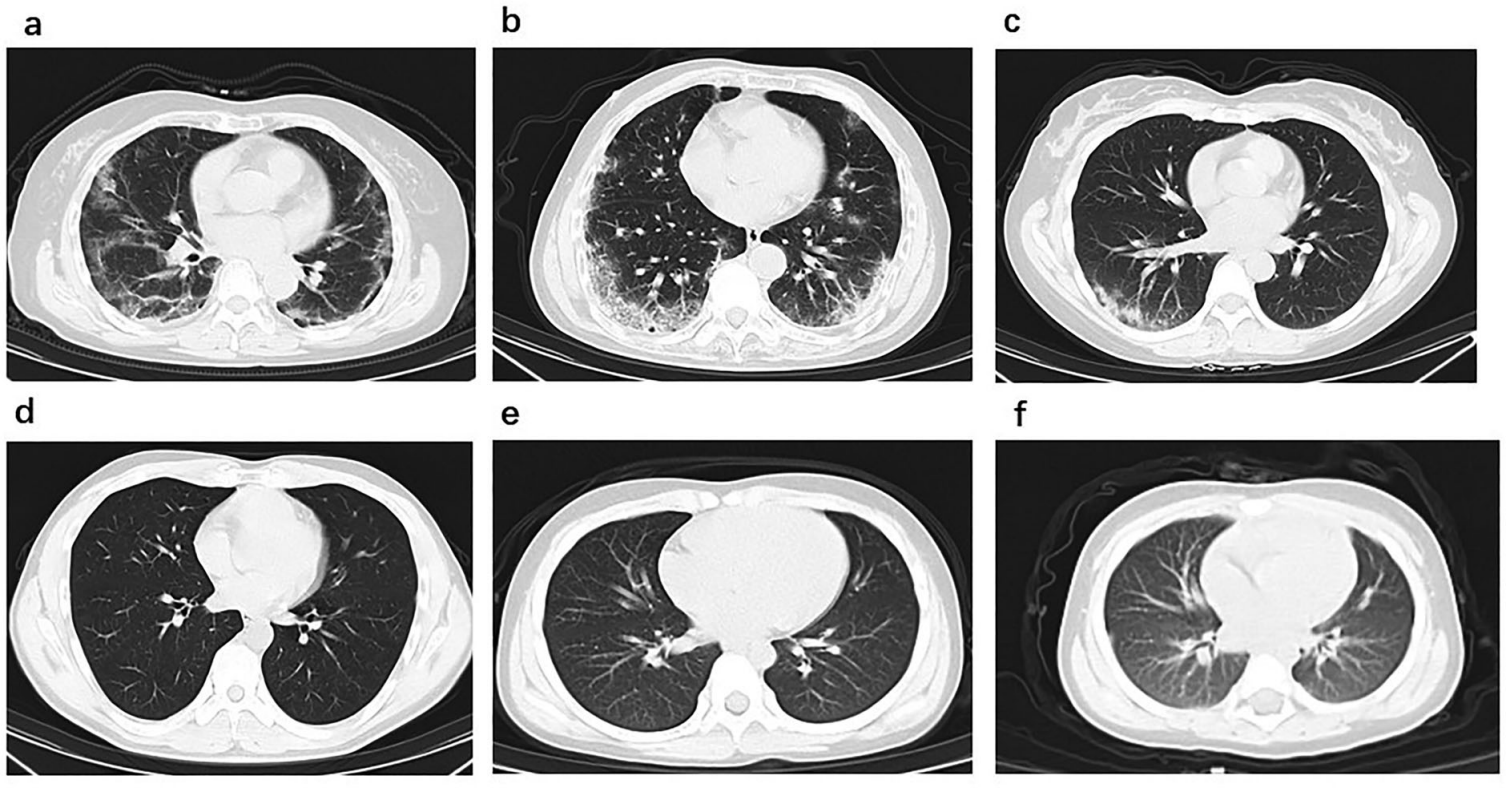
Chest CT images. (a) Transverse chest CT images from Case 1 showing bilateral ground-glass opacity, subsegmental areas of consolidation and subpleural line. (b) Transverse chest CT images from Case 2 showing peripheral pulmonary parenchymal ground-glass and consolidative pulmonary opacities. (c) Transverse chest CT images from Case 3 showing subsegmental areas of ground-glass opacity and consolidation. Transverse chest CT images from Case 4 (d), Case 5 (e) and Case 6 (f) were normal.

In infection control purpose, we recruited their four family members as COVID-19 close-contacts for COVID-19 screening, including Case 1’s daughter (Case 3), her son in law (Case 4), her grandson (Case 5) and granddaughter (Case 6), all of them lived together under one roof in last 14days. All SARS-CoV-2 RT-PCR assays of the four close-contacts’ nasopharyngeal swab specimens showed negative result. However, chest CT images of Case 3 (Figure 1c) showing local patchy shadowing indicated viral pneumonia, while chest CT images of other three close-contacts were normal (Figure 1d, 1e, 1f).

In concern of false-negative RT-PCR results, the family members were kept in Fever Clinic of PUMCH for further investigation. SARS-CoV-2-specific immunoglobin M (IgM) screening testing by gold immunochromatography assay (Hotgen Biotech Co., Ltd., Beijing, China) was immediately performed in the clinical laboratory, which reported positive for five of the six family members except Case 4. Follow-up enzyme-linked immunosorbent assay (ELISA, developed by Institute of Pathogen Biology, Chinese Academy of Medical Sciences & Peking Union Medical College) test confirmed SARS-CoV-2-specific positive IgM results for the five family members, and Case 2 also present SARS-CoV-2-specific immunoglobin G (IgG) positive. However, the repeated RT-PCR assays on the second day for five family members only clarified one more positive result for asymptomatic Case 5. The detail information of this family cluster are showed in Table 1.

**Table 1.** Clinical characteristics, chest CT features and laboratory findings of the family cluster.

	Case 1	Case 2	Case 3	Case 4	Case 5	Case 6
Family relationship	Wife	Husband	Daughter	Son in law	Grandson	Granddaughter
Epidemiological history						
Recent residency in Wuhan	Y	Y	N	N	Y	N
Date of leaving Wuhan	Jan 22	Jan 22	NA	NA	Jan 22	NA
Symptoms						
Date of initial symptoms	Feb 3	Feb 2	Feb 3	NA	NA	NA
Fever (maximum temperature)	38.0°C	37.6°C	36.4°C	36.6°C	36.4°C	36.1°C
Oxygen saturation	95%	97%	99%	100%	100%	98%
Nasal congestion	N	Y	N	N	N	N
Cough	Y	Y	Y	N	N	N
Laboratory examination						
White blood cell count (10⁹/L); (normal range 3.5-9.5)	5.01	5.11	5.16	9.83	5.85	9.72
Neutrophil count (10⁹/L); (normal range 2.0-7.5)	2.00	3.10	3.82	7.12	2.22	3.80
Lymphocyte count (10⁹/L); (normal range 0.8-4.0)	2.68	1.44	1.08	2.25	3.27	5.01
Chest CT images	Manifestation of viral pneumonia	Manifestation of viral pneumonia	Manifestation of viral pneumonia	Normal	Normal	Normal
SARS-CoV-2 RT-PCR assay§	Pos	Neg	Neg	Neg	Neg	Neg
SARS-CoV-2 RT-PCR assay after 24 h §#	ND	Neg	Neg	Neg	Pos	Neg
SARS-CoV-2-specific IgM (GICA)	Pos	Pos	Pos	Neg	Pos	Pos
SARS-CoV-2-specific IgM (ELISA)	Pos	Strong pos	Pos	Neg	Weak pos	Pos
SARS-CoV-2-specific IgG (ELISA)	Neg	Strong pos	Neg	Neg	Neg	Neg
Diagnosis	Confirmed COVID-19	Suspected COVID-19 patient*	Suspected COVID-19 patient*	COVID-19 close contact	Confirmed COVID-19	COVID-19 close contact

§Molecular assays were performed with two different SARS-CoV-2 RT-PCR kits simultaneously.

#If the result of the result of SARS-CoV-2 RT-PCR assay was negative, nasopharyngeal swabs were collected 24 h later for a second molecular assays.

*This reflected diagnosis on February 6, 2020. Follow-up molecular testing was positive for case 2 five days later and for case 3 one month later, respectively, and made COVID-19 diagnosis confirmed.

GICA= gold immunochromatography assay. ELISA= enzyme-linked immunosorbent assay. NA=not available. ND=not done. Y=Yes. N=No. Pos =positive. Neg =negative.

At last, two patients (Case 1 and Case 5) were diagnosed with COVID-19, two (Case 2 and Case 3) were suspected of COVID-19, and the four patients were transmitted to designated hospital in Beijing for further treatment. Case 4 and Case 6 were defined as COVID-19 close-contact for further isolation and observation at quarantine stations. Follow-up molecular testing was positive for Case 2 five days later and for Case 3 one month later, respectively, and made COVID-19 diagnosis confirmed.

For epidemiological analysis, although Case 1 was the proband of this family cluster, we speculated that Case 2 was the initial patient in this family cluster, based on the early symptom onset time, positive SARS-CoV-2-specific IgG antibodies and typical manifestation of viral pneumonia on chest CT images. Moreover, the positive result of the SARS-CoV-2-specific IgM antibody of Case 6 indicated that the subclinical or asymptomatic infection of COVID-19 could not be excluded.

Because of highly transmissible characteristic of COVID-19 [6], timely diagnosis and management of COVID-19 patients is essential. However, the diagnosis process of this family cluster cases reflected puzzled capacity of molecular assays in COVID-19 from first-line clinicians, although molecular assays was still “gold-standard” in relevant case diagnosis. It has been reported that upper respiratory tract samples for molecular testing is unreliable [5], and viral load may be significantly decreased in these samples since days onset [7]. In addition, acquirement of lower respiratory tract samples (e.g. sputum, endotracheal aspirate or bronchoalveolar lavage) as recommended were not practical in out-patient settings for patient screening. In view of control for highly transmittable pathogen like SARS-CoV-2, leading to great threaten to public health, it is urgently needed to develop effective complementary testing for COVID-19.

Although WHO only recommends serology testing as a “second-line” test where molecular assays is not available [1], the utility of serology should be more emphasized in the COVID-19 pandemic. Since early March, serology testing has been involved in updated China national guidelines as a direct evidence for COVID-19 diagnosis. Coupled with manifestation of viral pneumonia on chest CT images, or close-contact history of COVID-19 confirmed cases, a positive IgM results could suggest highly possibility of SARS-CoV-2 infection, and implementation of isolation and follow-up treatment should be initiated. Repeated RT-PCR assays and serology testing should be performed for patients with IgM positive, while a dynamic change of SARS-CoV-2-specific IgG from negative to positive supporting the COVID-19 diagnosis. For SARS infection[8] and SARS-Cov-2 infection[5], IgM seroconversion took place in acute infection period, while IgG could be detected later on, generally within a week. In addition, the turn-around-time for SARS-CoV-2 serology testing were generally only 15 min to 1 h, and the assay could be equipment-free (e.g. by immunochromatographic assay) or high-throughput (e.g. based on chemiluminescence platform). Today, spread trends of SARS-CoV-2 have emerged global-wide, and most countries are for large amount molecular screening for potential COVID-19 patients. In this occasion, serology testing is supposed to be a more efficient and cost-effective solution to deal with the situation.

In conclusion, our results emphasized the significance of serology testing to assist timely diagnosis of SARS-CoV-2 infections, especially for COVID-19 close contacts screening. Serology testing will be an effective tool for COVID-19 screening and be a powerful complementary diagnostic testing for diagnosis of COVID-19.
